# Biosimilar versus branded enoxaparin to prevent postoperative venous thromboembolism after surgery for digestive tract cancer: Randomized trial

**DOI:** 10.1371/journal.pone.0293269

**Published:** 2023-11-01

**Authors:** Chadli Dziri, Wafa Ben Hmida, Wejih Dougaz, Mehdi Khalfallah, Imen Samaali, Hichem Jerraya, Ibtissem Bouasker, Ramzi Nouira

**Affiliations:** 1 Tunis Medical School, Tunis El Manar University, Tunis, Tunisia; 2 Honoris Medical Simulation Center, Tunis Montplaisir, Tunisia; 3 Department B of General Surgery, Charles Nicolle’s Hospital, Tunis El Manar University, Tunis, Tunisia; Hospital Nossa Senhora da Conceição de Porto Alegre, BRAZIL

## Abstract

Cancer and/or major surgery are two factors that predispose to post-operative thrombosis. The annual incidence of venous thromboembolic disease (VTED) in cancer patients was estimated at 0.5%-20%. Surgery increases the risk of VTED by 29% in the absence of thromboprophylaxis. Enoxaparin is a low molecular weight heparin that is safe and effective. Branded Enoxaparin and biosimilar Enoxaparin are two enoxaparin treatments. This study aimed to compare Branded Enoxaparin with biosimilar Enoxaparin in patients operated on for digestive cancer regarding the prevention of postoperative thrombosis event, to compare the tolerance of the two treatments and to identify independent predictive factors of thromboembolic incident. A randomized controlled trial conducted in a single-centre, surgical department B of Charles Nicolle Hospital, over a 5-year period from October 12^th^, 2015, to July 08^th^, 2020. We included all patients over 18 who had cancer of the digestive tract newly diagnosed, operable and whatever its nature, site, or stage, operated on in emergency or elective surgery. The primary endpoint was any asymptomatic thromboembolic event, demonstrated by systematic US Doppler of the lower limbs on postoperative day 7 to day 10. The sonographer was unaware of the prescribed treatment (Branded Enoxaparin [BE] or biosimilar Enoxaparin [BSE]). Of one hundred sixty-eight enrolled patients, six patients (4.1%) had subclinical venous thrombosis. Among those who had subclinical thrombosis, four patients (5.6%) were in the Branded Enoxaparin group and two patients (2.7%) in the Biosimilar Enoxaparin group without statistically significant difference (p = 0.435). Analysis of the difference in means using Student’s t test demonstrated the equivalence of the two treatments. Our study allowed us to conclude that there was no statistically significant difference between Branded Enoxaparin and Biosimilar Enoxaparin regarding the occurrence of thromboembolic accidents postoperatively. BE and BSE are equivalent.

**Trial registration. Trial registration:** The trial was registered on CLINICALTRIALS.GOV under the number NCT02444572.

## Introduction

Venous thromboembolic disease (VTED) refers to a condition characterized by the formation of blood clots within the veins, commonly has two main clinical presentations: deep vein thrombosis (DVT) and/or pulmonary embolism (PE) [1]. The annual incidence of VTED in cancer patients is estimated between 0.5–20% [2], compared with 0.1% in the general population [3]. It is associated with a high morbidity and mortality [4]. Indeed, thromboembolic disease is the second leading cause of death in cancer patients [5]. VTED constitutes a financial burden to health care [6]. This high cost is related to the following: more consultations, three times more hospitalizations and a longer hospital stay [6].

The high risk of postoperative thrombosis is due to the combination of two risk factors: cancer and major surgery [7]. Indeed, cancer constitutes a state of acquired hypercoagulability due to the multiple relationships between this disease, the inflammation and haemostasis systems [8]. In surgical patients, the incidence of VTED is 29% in the absence of thromboprophylaxis [9]. This is due to vascular injury, immobilization, and venous stasis [10]. Multiple risk factors have been identified such as: the nature of the cancer, the stage of the cancer and associated treatments (radiotherapy, chemotherapy) [11].

Several prospective randomized studies conducted in the early 1980s and late 1990s showed that thromboprophylaxis reduces the risk of thrombosis compared to no prophylaxis or placebo [12–14]. Thromboprophylaxis also reduced the overall cost of health care [15].

Over the years, several molecules have been introduced on the market: unfractionated heparin (UFH), low molecular weight heparin (LMWH), vitamin K antagonist (VKA) and direct oral anticoagulants (DOACs). Several guidelines have recommended LMWH or UFH in major abdominopelvic surgery in the absence of bleeding risk [16–18]. According to international clinical practice guidelines, LMWH is still advocate for the treatment and the prevention of cancer associated thrombosis [18].

LMWH has advantages over UFH such as a longer half-life and predictable bioavailability [10]. In addition, LMWH is less restrictive to prescribe with a single injection per day compared to two or three injections per day for UFH [16]. A Meta-Analysis compared the efficacy and safety of three types of parenteral anticoagulants for the initial treatment of VTE in people with cancer. It pooled five studies (inclusion of 418 patients with cancer), showed a significant reduction in mortality with LMWH compared with UFH [19]. Enoxaparin is a widely used, safe and effective low molecular weight heparin [20]. Original Branded Enoxaparin (BE) was granted marketing authorization (MA) in 1990. Biosimilar Enoxaparin (BSE) was granted marketing authorization in 2007. It should be noted that BSE is less expensive than BE.

Several biosimilar versions of Lovenox^®^ (Sanofi, Paris, France), the original branded form of enoxaparin, have become available for clinical use for the indications that Lovenox^®^ was previously approved [21].

This study aimed to compare a Biosimilar Enoxaparin [BSE] with Branded Enoxaparin [BE] in patients undergoing digestive cancer surgery in the prevention of venous thrombosis in the postoperative period, to compare the safety of the two treatments and to identify the factors predictive of thrombo-embolic event.

## Materials and methods

This is a prospective, comparative, randomized study, conducted in a single centre during four years and nine months: from October 12, 2015, to July 08, 2020.

1. Patients:

Included patients were: 1) over 18 years of age with proven, known or newly diagnosed, operable digestive cancer regardless of its nature, location, or stage, 2) and all patients who underwent emergency or elective surgery. Non-included patients were: 1) participating in another study, 2) patients with prior unfractionated heparin impregnation in the last 30 days, 3) patients with chronic renal failure with creatinine clearance<30 ml/min, 4) patients with a known history of peripheral and/or deep vein thrombosis occurring within three months prior to study inclusion, 5) patients under anticoagulation treatment within the last three months, 6) patients with a known disorder of haemostasis, 7) patients unwilling to participate in this study or unable to understand its objectives, 8) and patients with a pregnancy occurring during the study were not included. The follow-up of the included patients was done according to the CONSORT guidelines [22]. Randomization was managed by an Interactive Web-Response System (IWRS), using the randomization module of a validated electronic data capture system (DACIMA). The system provided concealed random allocation sequences. The group allocation is set automatically when the eligibility criteria are verified, and when the patient is available to be randomized. The IWRS provided allocated open labeled treatment to the site coordinator/investigator. Actual treatment is captured in the system and compared automatically with the concealed sequence. Any discrepancies in actual treatment allocation are considered as randomization error, and they are reported in the Case Report Form (CRF). As this is an open label randomized clinical trial, treatment blindness is set to the Doppler Ultrasounds rater.

2. Disease:

Digestive cancers (oesophagus, stomach, small bowel, colon, rectum, hepatobiliary and pancreas) in an early or advanced stage, complicated or not, were included. Extra-digestive cancers of the abdomen and patients with tumours without histological confirmation were not included.

3. Treatment:

According to randomization the patient received Branded Enoxaparin (BE) (Lovenox^®^, Sanofi—Aventis Pharmaceuticals, France) 4000 IU one injection per day for 30 days or Biosimilar Enoxaparin (BSE) (Enoxa^®^, Medis Pharmaceuticals, Tunisia) 4000 IU one injection per day for 30 days. The injections were administered subcutaneously eight to 12 hours after the surgery.

4. Outcome measures:

The primary endpoint was any asymptomatic thromboembolic event, assessed by a systematic Doppler ultrasound of the vessels of the lower limbs between day-7 and day-10 post surgery. This evaluation was done in a blinded fashion. The sonographer was not aware of the patients’ randomization. The secondary endpoints were the occurrence of a symptomatic thrombotic event, the occurrence of incident heparin-induced thrombocytopenia, the occurrence of bleeding events and mortality.

5. Calculation of sample size and randomization

The occurrence of subclinical thrombosis postoperatively after digestive cancer surgery is estimated to be 18% [8]. Population size estimation was based on the P_1_-P_2_ thrombosis frequency difference equivalence procedure. P_1_ is the frequency in the BSE group and P_2_ is the frequency in the comparator BE group. The sample size estimation required 70 subjects per arm, with a two-sided alpha level of 5% and a power of 80%, and with margin error for mean difference ranging from -0.20 to +0.20. Sample size was overestimated by 10% as per loss of follow-up and/or data discrepancies expectation. An overall sample size of 160 subjects was planned to be enrolled in the study. Sample size estimation used the Power and Sample Size Software (PASS v2008). Allocated study subjects were randomly assigned by an interactive web-response system (DACIMA), using simple 1:1 non-stratified sequence and a block size of 4.

The database used the same validated electronic data capture system (DACIMA), which complies with FDA 21 CFR part 11 (Food and Drug Administration 21 Code of Federal Regulations part 11), HIPAA (Health Insurance Portability and Accountability Act) & ICH (International Conference on Harmonization) requirements.

6. Compliance, Protocol Violation, Patient follow-up

Compliance was defined as the number of days enoxaparin was taken. Days not taken were reported in relation to the previously planned number of days of intake and were secondarily translated into percentage. It was considered "nonadherence" if the rate of non-take days ≥20%. Protocol violation was defined by any selected patient who had "nonadherence" to study treatment ≥20%, randomization allocation error, and Doppler ultrasound performed outside the 7- to 10-day postoperative interval.

Several follow-up visits were performed. An inclusion visit verified the eligibility of patients and included them if they met the selection criteria. The patient is hospitalized for seven to 10 days. Ultrasound and clinical control were performed between post-operative day seven to 10.

A 30-day follow-up visit for a clinical check-up and closure of the study was planned for each patient.

7. Data collection:

Eighty eight variables were culled, divided into: 1) Inclusion criteria: age over 18 years, proven cancer, indication for surgical treatment, preventive administration of Enoxaparin BSE or BE, 2) Demographic variables: age, gender, weight, height, body mass index (BMI), comorbidities (history of thrombosis, diabetes, cardiovascular disease, respiratory failure, renal failure, corticosteroid therapy, immunosuppression, polycythaemia, postpartum); 3)Preoperative variables: date of disease discovery, nature of the cancer, site, extension work-up, biology, preoperative chemotherapy, preoperative radiotherapy, 4) Intraoperative variables: operative time, nature of the procedure, type of anaesthesia, intraoperative bleeding; and 5) Postoperative variables: treatment (dose, date of administration, duration), biological follow-up during the study, Doppler ultrasound (date, result), subclinical and clinical thromboembolic events, bleeding event, heparin-induced thrombocytopenia, death, follow-up visit (1 month), All data have been collected by WD and WBH.

8. Statistical analysis:

All data were entered into SPSS® statistical software (IBM Corp. Released 2017. IBM SPSS Statistics for Windows, Version 25.0. Armonk, NY: IBM Corp.).

Qualitative variables were expressed by their frequency and percentages. Quantitative variables were mentioned by the mean and standard deviation when the distribution was Gaussian and by the median with extremes and interquartile range when the distribution variable was not Gaussian.

The comparison of BSE versus BE groups was performed by independent groups Student’s t-test or the non-parametric Mann-Whitney U test for continuous variables. Categorical variables were compared by Pearson’s chi-squared test or Two-sided Fisher’s exact test, when appropriate. The margin of equivalence, estimated as the difference in frequencies, was between -0.20 and +0.20 (difference in proportion is 0.20). The real difference would be 0.00. The calculation assumes the performance of a one-sided Student’s t-test. The 95% confidence intervals were compared graphically with the equivalence margin interval. The value of p≤0.05 was considered the threshold for significance.

We performed a prognostic study to identify independent predictors of clinical and subclinical thrombosis. We performed a crude bivariate analysis using appropriate statistical tests. Factors that were accompanied by a value of p≤0.05 were entered into a Backward logistic regression model. Each retained factor was accompanied by its relative risk and 95% confidence interval with the p value.

9. Disclosure, Trial registration

The Médis laboratory provided the BE treatment and sponsored the practice of Doppler ultrasound exams. The study was carried out in accordance with the current version of the Helsinki Declaration (52nd WMA General Assembly, Edinburgh, Scotland, October 2000). The clinical trial was conducted in accordance with the guidelines of the International Conference on Harmonization (ICH) on Good Clinical Practice (GCP). All patients provided written informed consent to participate in the study before being included.

The patient information sheet details the procedures involved in the study (objectives, methodology, potential risks, expected benefits) and the investigator explains them to each patient. The patient signed the consent form to indicate that the information had been explained and understood. The patient was then given time to review the information presented before signing and dating the informed consent form to indicate that he fully understood the information and volunteered to participate in the study. The trial was registered on CLINICALTRIALS.GOV under the number NCT02444572.

10. Practical conduct:

After patient’s admission in the surgical department B of Charles Nicolle Hospital, a number was assigned to the medical record. The intern in charge of the patient wrote the patient’s medical record, specifying the address and telephone number, under the supervision of the resident and the senior physician in charge. Once it was verified that the patient was eligible to be included in the study, a balanced randomization was done on the same day using the DACIMA software. The person in charge of randomization was CD (one of the authors). The treatment was started eight hours after the end of the operation if there was no bleeding.

Once the ultrasound was done between the seventh and 10th postoperative day, the patient was discharged except when he/she had complications requiring a prolonged hospitalization. The randomization is set automatically by the validated IWRS (DACIMA tool). The allocation sequence was designed at the CRF development step before study kick-off. The data-manager who performed the CRF building, and the randomization sequence allocation wasn’t involved in the study management. The data collection at site level was performed by study coordinators and investigators who were aware about the allocated treatment after running the IWRS, when eligibility criteria were verified, and the subject had been available for randomization. As the protocol was an open labeled randomized, rater blind, clinical trial, only the ultrasounds specialist was unaware about the treatment allocation. The person performed the randomization were involved in the data collection.

## Results

### Comparability of groups

**Fig 1:**
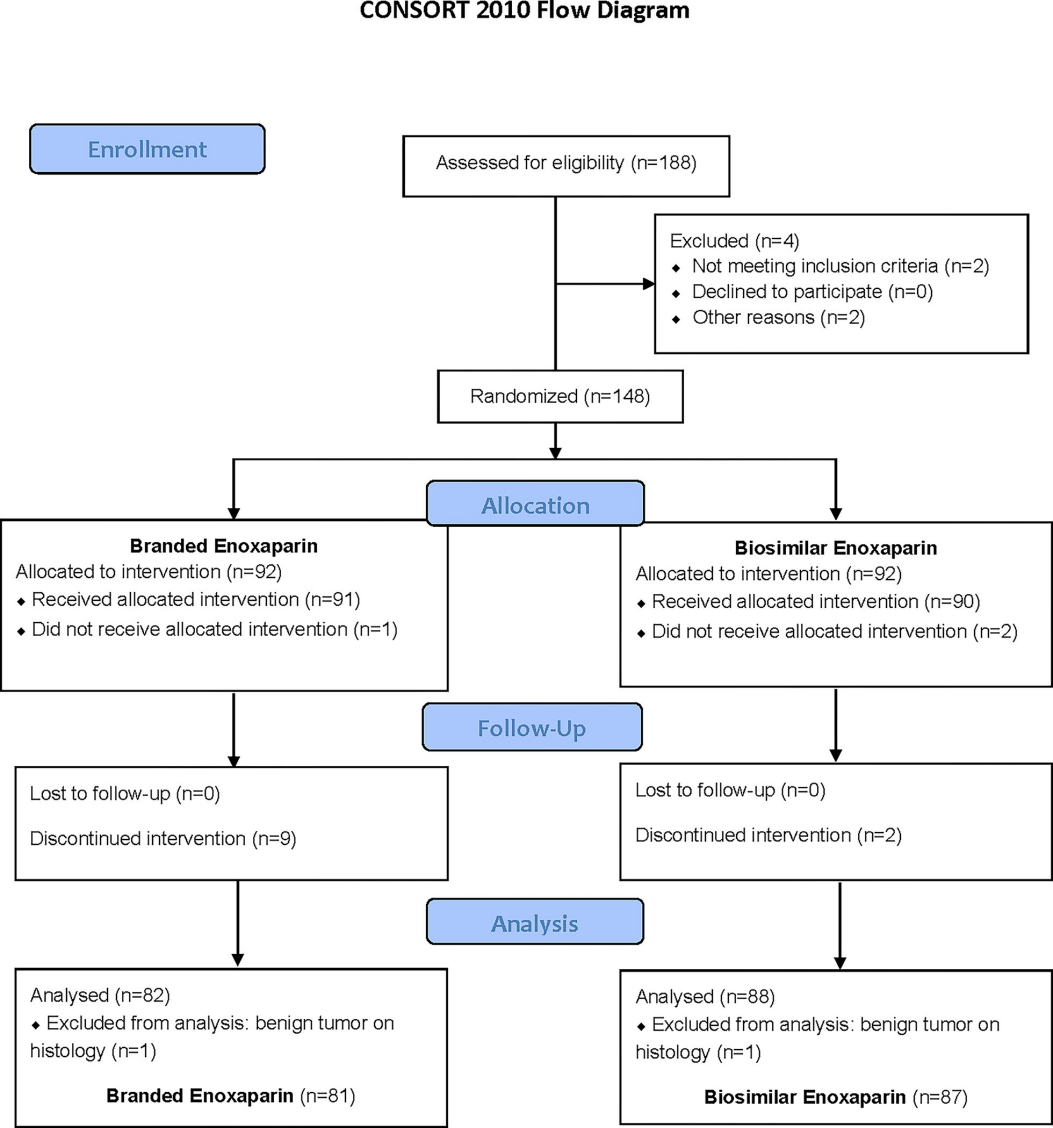
Screening, randomization, and follow-up of study participants.

Over four years and nine months, 168 eligible patients, had been operated in the surgical department B of Charles Nicolle Hospital for digestive cancer. Of 188 patients enrolled to participate in the study (Fig 1), two patients did not meet the inclusion criteria and two others were not randomized. One hundred and eighty-four patients were randomized. Ninety-two in the BE group and 92 in the BSE group. After allocation, three patients did not receive their treatment due to an inclusion error. One patient was in the BE group and two patients were in the BSE group. During follow-up, nine patients in the BE group had premature discontinuation of treatment. The causes were renal failure, cardiac decompensation with initiation of treatment with Acenocoumarol (Sintrom^®^, Novartis, France) noncompliance with protocol and death on the first postoperative day owing to multiple organ failure. Two patients in the BSE group had premature discontinuation of treatment due to protocol violations. During the analysis, two patients were excluded because there were no cancers according to the pathology of the surgical specimen. One patient was in the BE group and one patient was in the BSE group. In practice, after the elimination of randomization errors, protocol violation, patients with incomplete follow up, full data were available for 168 patients (81 in the BE group; 87 in the BSE group). Fig 1 reported the CONSORT 2010 flow diagram of patients: Screening, Randomization, and Follow-up of Study Participants.

No statistically significant difference was found in patients demographics and thrombosis factors (Table 1).

**Table 1:** Comparative study: Demographic variables (branded enoxaparin vs biosimilar enoxaparin groups).

		BE	BSE	P
		(N = 81)	(N = 87)	-
**Age #**	Mean ± SD*	59.9± 11.8	60.3± 12.9	-
**Gender $** Male Female		45 (55.6%) 36(44.4%)	39(44.8%) 48(55.2%)	-
**Ascites $ $**	Yes No	3 (3.7%) 78(96.3%)	0(0%) 87(100%)	-
**Peritoneal carcinomatosis $ $**	Yes No	1(1.2%) 80 (98.8%)	0 (0%) 87 (100%)	-
**Portal hypertension $ $**	Yes No	1 (1.2%) 80 (98.8%)	0 (0%) 87 (100%)	-
**Factors affecting wound healing $**	Yes No	6 (7.4%) 75 (92.6%)	14 (16.1%) 73 (83.9%)	-
**Corticosteroid for 6 months $ $**	Yes No	0 (0%) 81 (100%)	1 (1.1%) 86 (98.9%)	-
**Reduced mobility/Bed rest >4j $ $**	Yes No	2 (2.5%) 79 (97.5%)	0 (0%) 87 (100%)	-
**Varicose veins $ $**	Yes No	0 (0%) 81 (100%)	1 (1.1%) 86 (98.9%)	-
**Ischemic stroke $ $**	Yes No	1 (1.2%) 80 (98.8)	1 (1.1%) 86 (98.9%)	-
**Hormonal treatment of postmenopausal $ $**	Yes No	0 (0%) 81 (100%)	1 (1.1%) 86 (98.9%)	-
**Postpartum $ $**	Yes No	0 (0%) 81 (100%)	1 (1.1%) 86 (98.9%)	-
**Polyglobulia $ $**	Yes No	0 (0%) 81 (100%)	1 (1.1%) 86 (98.9%)	-
**Cardiovascular history $**	Yes No	28 (34.6%) 53 (65.4%)	29 (33.3%) 58 (66.7%)	-
**Unstable angina $ $**	Yes No	0 (0%) 81 (100%)	1 (1.1%) 86 (98.9%)	-
**Respiratory history $ $**	Yes No	1 (1.2%) 80 (98.8%)	1 (1.1%) 86 (98.9%)	-
**Diabetes $**	Yes No	20 (24.7%) 61 (75.3%)	22 (25.3%) 65 (74.7%)	-
**History of immunosuppression $ $**	Yes No	0 (0%) 81 (100%)	1 (1.1%) 86 (98.9%)	-

* SD: Standard Deviation BE: Branded Enoxaparin BSE: Biosimilar Enoxaparin.

# Student’s t-test or ## the non-parametric Mann-Whitney U test used for comparison of continuous variables.

Categorical variables were compared by $ Pearson’s chi-squared test or $ $ Two-sided Fisher’s exact test, when appropriate.

There was also no statistically significant difference between the two groups for the pre and intraoperative variables (Table 2). The most frequent sites of cancer were colon and rectum (71.6%) for BE group, and 66.7% for BSE group (p = 0.742). Seventeen patients (21%) received preoperative chemotherapy in the BE group and twenty-four patients (27.6%) in the BSE group (p = 0.320). Ten patients (12.3%) received preoperative radiotherapy in the BE group and eighteen patients (20.7%) in the BSE group (p = 0.147) (Table 2). Most patients, operated on, had T3 or T4 cancers with 54 classified T3 or T4 (80.6%) in the BE group and 54 classified T3 or T4 (74%) in the BSE group without statistically significant difference between the two groups (p = 0.306). The majority had positive nodes with 34 positive nodes (50.7%) in the BE group and 42 positive nodes (57.5%) in the BSE group (p = 0.421). Seven patients (8.6%) had metastases in the BE group and nine patients (10.3%) in the BSE group (p = 0.707) (Table 2).

**Table 2:** Comparative study: Pre and intraoperative variables (branded enoxaparin vs biosimilar enoxaparin groups).

		BE	BSE	p
		(N = 81)	(N = 87)	
**Tumor site $** • Esophagus, Stomach Small intestine • Colon, Rectum • Liver, bile ducts, Pancreas		14 (17.3%) 58 (71.6%) 9 (11.1%)	19 (21.8%) 58 (66.7%) 10 (11.5%)	0.742
**Nature $ $** Primitive	Yes No	81 (100%) 0 (0%)	86 (98.9%) 1 (1.1%)	1.000
**Metastasis $**	Yes No	7 (8.6%) 74 (91.4%)	9 (10.3%) 78 (89.7%)	0.707
**Biology** ## Creatinine (μmol/l) Clearance (ml/min) Hemoglobin (g/dl) Red blood cells (10^9^/microlitre) Platelets (10 ^3^ e/mm3) Hematocrit (%)	Median [ext] Median [ext] Median [ext] Median [ext] Median [ext] Median [ext]	67 [39 – 494] 100.8 [46.8–132.3] 11.6 [6.9–21.4] 4.4 [3.2–11.6] 287 [73–594] 35 [22.7–55]	63.5 [7.8–176] 101.7 [52.2–609.7] 11.5 [6.9–15.4] 4.4 [2.8–19.5] 273 [133–628] 35 [23–43]	0.349 0.419 0.604 0.583 0.738 0.782
**Preoperative chemotherapy $**	Yes No	17 (21%) 64 (79%)	24 (27.6%) 63 (72.4%)	0.320
**Preoperative radiotherapy $**	Yes No	10 (12.3%) 71 (87.7%)	18 (20.7%) 69 (79.3%)	0.147
**Duration of surgery ##**	Median [ext]	300 [90 .540]	300 [74. 540]	0.779
**Bleeding during Surgery $ $**	Yes No	0 (0%) 81 (100%)	1 (1.1%) 86 (98.9%)	1.000
**TNM** **Tumor (T) $** **Nodes (N) $** **Metastasis (M) $**	Tx/T0/T1/T2 T3/T4 N0/Nx N+ M0 M1	13 (19.4%) 54 (80.6%) 33 (49.3%) 34 (50.7%) 74 (91.4%) 7 (8.6%)	19 (26%) 54 (74%) 31 (42.5%) 42 (57.5%) 78 (89.7%) 9 (10.3%)	0.306 0.421 0.707

Median [ext] = Median [extreme].

# Student’s t-test or

## the non-parametric Mann-Whitney U test used for comparison of continuous variables.

Categorical variables were compared by $ Pearson’s chi-squared test or $ $ Two-sided Fisher’s exact test, when appropriate.

### Rate of thromboembolic events

Seventy-one patients (87.7%) in the BE group had an ultrasound scan during the study and seventy-four patients (85.1%) in the BSE group with no statistically significant difference between the two groups (p = 0.625) (Table 3). A total of 145 patients were systematically evaluated by Doppler ultrasound of the vessels of the lower limbs between day-7 and day-10 post-surgery. The sample size of 145 is in accordance with the calculation of the number of subjects needed.

**Table 3:** Comparative study: Post-operative variables (branded enoxaparin vs biosimilar enoxaparin groups).

		BE	BSE	p
		(N = 81)	(N = 87)	
**Echodoppler performed** $	Yes No	71 (87.7%) 10 (12.3%)	74 (85.1%) 13 (14.9%)	0.625
**Subclinical thromboembolic events** * $ $	Yes No	**4 (5.6%)** **67 (94.4%)**	**2 (2.7%)** **72 (97.3%)**	**0.435**
**Clinical and subclinical events** ** $ $	Yes No	7 (8.6%) 74 (91.4%)	6 (6.9%) 81 (93.1%)	0.672
**Post-operative complications** $	Yes No	14 (17.3%) 67 (82.7%)	8 (9.2%) 79 (90.8%)	0.120
**Post-operative bleeding** $ $	Yes No	2 (2.5%) 79 (97.5%)	1 (1.1%) 86 (98.9%)	0.610
**Transfusion** $ $	Yes No	2 (2.5%) 79 (97.5%)	1 (1.1%) 86 (98.9%)	0.610
**Thrombocytopenia** $ $	Yes No	0 (0%) 81 (100%)	1 (1.1%) 86 (98.9%)	1.000
**Death** $	Yes No	6 (7.4%) 75 (92.6%)	11 (12.6%) 76 (87.4%)	0.261
**Time of randomization ##**	Median IQR	0 [0–0]	0 [0–0]	0.837
**Time of Ultrasound** ##	Median IQR	7 [7 – 8]	7 [7–7.5]	0.683
**Time to onset of thrombosis** ##	Median IQR	8 [7 – 12]	9 [7 – 13]	0.160
**Time of death** ##	Median IQR	10 [6.25–14.25]	16 [8 – 24]	0.131
**Postoperative stay ##**	Median IQR	8 [7–13.5]	2 [7 – 13]	0.710

*23 ultrasounds not performed (12 deceased, nine left the hospital without performing a Doppler ultrasound, two had a pulmonary embolism recognized on CT scan).

** clinical and subclinical events: Six subclinical thromboses + seven clinical events (six pulmonary embolisms + one symptomatic thrombosis)–IQR (25%-75%): Interquartile range—BE: Branded Enoxaparin BSE: Biosimilar Enoxaparin.

# Student’s t-test or

**##** the non-parametric Mann-Whitney U test used for comparison of continuous variables.

Categorical variables were compared by $ Pearson’s chi-squared test or $ $ Two-sided Fisher’s exact test, when appropriate.

Of those who experienced subclinical thrombosis, four patients (5.6%) were in the BE group and two patients (2.7%) in the BSE group (p = 0.435) (Table 3). Therefore, there was no significant difference for the primary endpoint between the two groups. Clinical and subclinical events were distributed as follows: seven events (8.6%) in the BE group and six (6.9%) in the BSE group (p = 0.672) (Table 3). The median time to thrombosis was eight days [7–12] in the BE group and nine days [7–13] in the BSE group (p = 0.160) (Table 3).

**Fig 2:**
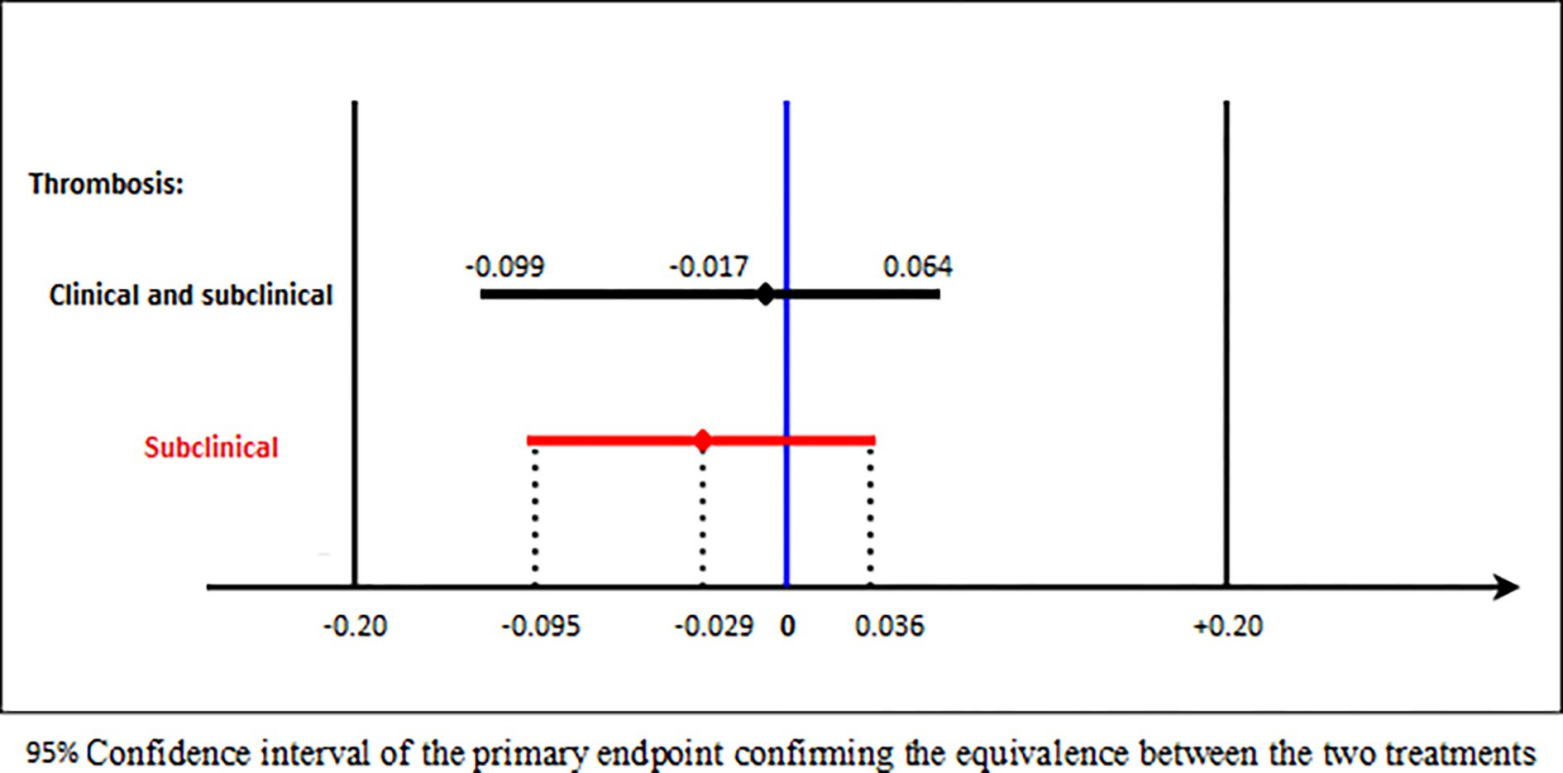
Equivalence between branded enoxaparin and biosimilar enoxaparin.

The analysis of the difference in means using Student’s t test showed that the interval of the difference in means was [-0.095 +0.036]. This interval was included in the interval [-0.20 +0.20] confirming that there was no significant difference between BSE and BE in preventing thromboembolic events as shown in Fig 2.

The number of patients who developed a post-operative complication was 14 (17.3%) in the BE group and eight (9.2%) in the BSE group (p = 0.120). Two patients (2.5%) had postoperative bleeding in the BE group and one patient (1.1%) had postoperative bleeding in the BSE group (p = 0.610). No incident of heparin-induced thrombocytopenia was mentioned in BE group and one incident occurred in BSE group (p = 1.000). There were six deaths (7.4%) in the BE group and 11 deaths (12.6%) in the BSE group without statistically significant difference between the two groups (p = 0.261). The median time to death was 10 days [6.25–14.25] in the BE group and 16 days [8 – 24] in the BSE group (p = 0.131) (Table 3).

### Risk factors of clinical and subclinical thrombosis events

The crude bivariate analysis showed no statistically significant difference between the two groups of patients regarding the occurrence of venous thrombosis postoperatively (Tables 4 and 5).

**Table 4:** Demographic variables predicting clinical and subclinical thrombosis (crude bivariate analysis).

Clinical and subclinical thrombosis				
		**Yes**	**No**	**p**
		(N = 13)	(N = 155)	
**Age #**	Mean ±SD	62.6 ±11.8	59.9 ±12.4	0.445
**Gender $** Male Female		6 (46.2%) 7 (53.8%)	78 (50.3%) 77(49.7%)	0.773
BMI* (n = 20) (Kg/m^2^) ##	Mean ± SD	20.5±3.6	24.8±3.9	0.165
**Smoking $ $**	Yes No	0(0%) 7 (100%)	14 (13.9%) 87 (86.1%)	0.591
**Ascites $ $**	Yes No	0 (0%) 13 (100%)	3 (1.9%) 152 (98.1%)	1.000
**Peritoneal carcinomatosis $ $**	Yes No	0 (0%) 13 (100%)	1 (0.6%) 154 (99.4)	1.000
**Portal hypertension $ $**	Yes No	0 (0%) 13 (100%)	1 (0.6%) 154 (99.4%)	1.000
**Factors affecting wound healing $ $**	Yes No	1 (7.7%) 12 (92.3%)	19 (12.3%) 136 (87.7%)	1.000
**Corticosteroid for 6 months $ $**	Yes No	0 (0%) 13 (100%)	1 (0.6%) 154 (99.4%)	1.000
**Reduced mobility/Bed rest >4j $ $**	Yes No	0 (0%) 13 (100%)	2 (1.3%) 153 (98.7%)	1.000
**Varicose veins $ $**	Yes No	0 (0%) 13 (100%)	1 (0.6%) 154 (99.4%)	1.000
**Ischemic stroke $ $**	Yes No	0 (0%) 13 (100%)	2 (1.3%) 153(98.7%)	1.000
**Hormonal treatment of postmenopausal $ $**	Yes No	0 (0%) 13 (100%)	1 (0.6%) 154 (99.4%)	1.000
**Postpartum $ $**	Yes No	0 (0%) 13 (100%)	1 (0.6%) 154 (99.4%)	1.000
**Hormonal treatment of postmenopausal $ $**	Yes No	0 (0%) 13 (100%)	1 (0.6%) 154 (99.4%)	1.000
**Postpartum $ $**	Yes No	0 (0%) 13 (100%)	1 (0.6%) 154 (99.4%)	1.000
**Polyglobulia $ $**	Yes No	0 (0%) 13 (100%)	1 (0.6%) 154 (99.4%)	1.000
**Cardiovascular history $ $**	Yes No	3 (23.1%) 10 (76.9%)	54 (34.8%) 101 (65.2%)	0.546
**unstable angina $ $**	Yes No	0 (0%) 13 (100%)	1 (0.6%) 154 (99.4%)	1.000
**Respiratory history $ $**	Yes No	1 (7.7%) 12 (92.3%)	1 (0.6%) 154 (99.4%)	0.149
**Diabetes $ $**	Yes No	2 (15.4%) 11 (84.6%)	40 (25.8%) 115 (74.2%)	0.521
**History of immunosuppression** $ $	Yes No	0 (0%) 13 (100%)	1 (0.6%) 154 (99.4%)	1.000

SD = standard deviation—BMI* (n = 20): Only 20 patients had available BMI.

# Student’s t-test or

## the non-parametric Mann-Whitney U test used for comparison of continuous variables.

Categorical variables were compared by $ Pearson’s chi-squared test or $ $ Two-sided Fisher’s exact test, when appropriate.

**Table 5:** Pre- and intra-operative variables predictive of clinical and subclinical thrombosis (crude bivariate analysis).

		Clinical and subclinical thrombosis		
		Yes	No	p
		(N = 13)	(N = 155)	
**Tumor site $** Esophagus, Stomach, Small intestine Colon, Rectum Liver, bile ducts, Pancreas		4 (30.8%) 8 (61.5%) 1 (7.7%)	29 (18.7%) 108 (69.7%) 18 (11.6%)	0.558
**Nature $ $** Primitive	Yes No	13 (100%) 0 (0%)	154 (99.4%) 1 (0.6%)	1.000
**Metastasis $ $**	Yes No	1 (7.7%) 12 (92.3%)	15 (9.7%) 140 (90.3%)	1.000
**Biology** # Creatinine (μmol/l) Clearance (ml/min) Hemoglobin (g/dl) Red blood cells (10^9^/microlitre) Platelets (10^3^ e/mm3) Hematocrit (%)	Mean ± SD Mean ± SD Mean ± SD Mean ± SD Mean ± SD Mean ± SD	65±14 53.8±2.2 10.8±2.5 5±5.4 304±120 34±6.8	72.1±43.4 131.2±126.1 11.5±2 5.2±2.7 293±105.8 35.1±5.35	0.886 0.143 0.182 0.968 0.061 0.674
**Preoperative chemotherapy $ $**	Yes No	1 (7.7%) 12 (92.3%)	40 (25.8%) 115 (74.2%)	0.191
**Preoperative radiotherapy $ $**	Yes No	1 (7.7%) 12 (92.3%)	27 (17.4%) 128 (82.6%)	0.697
**Duration of surgery #**	Mean ± SD	324.6±114.6	307.1±112.7	0.446
**Bleeding during surgery $ $**	Yes No	0 (0%) 13 (100%)	1 (0.6%) 154 (99.4%)	1.000
**TNM** **Tumor (T) $ $** **Nodes (N) $ $** **Metastasis (M) $ $**	Tx/T0/T1/T2 T3/T4 N0/Nx N+ M0 M1	2 (20%) 8 (80%) 3 (30%) 7 (70%) 12 (92.3%) 1 (7.7%)	30 (23.1%) 100 (76.9%) 61 (46.9%) 69 (53.1%) 140 (90.3%) 15 (9.7%)	1.000 0.345 1.000

SD = standard deviation.

# Student’s t-test or

## the non-parametric Mann-Whitney U test used for comparison of continuous variables.

Categorical variables were compared by $ Pearson’s chi-squared test or $ $ Two-sided Fisher’s exact test, when appropriate.

There were no predictors of postoperative thrombosis after digestive oncology surgery in this study.

## Discussion

Our randomized clinical trial showed that there was no statistically significant difference between BE and BSE groups in the occurrence of postoperative thromboembolic events. There was no statistically significant difference between BE and BSE groups regarding incident heparin-induced thrombocytopenia. No predictors factors of postoperative thrombosis after digestive oncology surgery were identified.

Our RCT is the first to compare BE with BSE on an asymptomatic thromboembolic event as the primary end point, while controlling for adverse events.

A biosimilar drug is a biological drug with the same qualitative and quantitative composition in active substance and the same pharmaceutical form as a reference biological drug. A biosimilar is not regarded as a generic of a biological medicine [23]. This is mostly because the natural variability and more complex manufacturing of biological medicines do not allow an exact replication of the molecular microheterogeneity [23]. Biosimilar drugs were more difficult to develop than generics and this was even more valid for low molecular weight heparins [24]. The rigorous chemical analyses will detect impurities such as the presence of inactive components not found in the original product leading to heparin-induced thrombocytopenia [25]. Nacho and Rosa [26] stated that biosimilar and biological reference medicines are similar but not identical. Therefore, randomized clinical trials still play an indispensable role in evaluating the efficacy and safety of a treatment [27,28] including Generic and biosimilar products. Imberti et al [29] concluded that to prove the efficacy and safety of a LMWH biosimilar, at least one randomized comparative study should be conducted. This study should preferably concern the prevention of venous thrombosis, in a population at high thrombo-embolic risk [29]. Indeed, safety considerations in the development of biosimilars was at the centre of regulatory guidelines [30].

A systematic review, reported by Mielke et al in 2018 [31], confirmed that the high variability of the submitted studies is still present. The authors [31] insisted that sponsors are asked to show equivalence and not non-inferiority. Our study’s design was a randomized trial for equivalence. Indeed, the equivalence of the two treatments (BSE versus BE) in our RCT was shown by the confidence interval of the difference in means [-0.095 +0.036] which is included in the equivalence interval [-0.20 +0.20].

As concerns Generic, several randomized controlled trials (RCT) [32–35] reported a comparison between two different enoxaparin products (Generic versus branded enoxaparin). One RCT [32] concluded that generic and branded enoxaparin had similar clinical efficacy and safety outcomes. Two RCT [33,34] concluded that generic LMWH was safe and effective as the branded enoxaparin to prevent VTED. Another RCT [35] compared generic and branded subcutaneous enoxaparin, the authors showed a bioequivalence between the two formulations which were well tolerated. Furthermore, a RCT [36] compared a generic with the originated LMWH, it showed a bioequivalence between the two formulations regarding the LMWH’s influence on coagulation tests.

As concerns biosimilar, two RCTs compared new biosimilar treatment of Enoxaparin to a reference treatment [37,38]. One RCT [37] showed that the biosimilar was equivalent to the reference enoxaparin in all primary and secondary parameters, as required by the European Medicines Agency to grant marketing authorization [37]. A RCT [38] of non-inferiority, phase III clinical study was designed to evaluate the efficacy and safety of enoxaparin Cristalia in comparison to branded enoxaparin for the prophylaxis of VTED in patients undergoing abdominal surgeries with a high risk for the development of thromboembolic disease [38]. Enoxaparin Cristalia was as effective as branded enoxaparin to prevent VTED [38]. In 2020, Qneibi et al. [21] concluded that Heparinox (a new biosimilar) was bioequivalent to the original branded enoxaparin based upon *in vitro* tests. In 2021, Fantoni et al. [39] conducted an observational, retrospective study to assess the safety and effectiveness of Inhixa in preventing VTED in medical and surgical inpatients. They concluded to the safety and effectiveness of biosimilar enoxaparin (Inhixa) to prevent VTED [39]. Our study was similar to Ramacciotti’s study [37] in terms of endpoints, study population and doses administered. However, they had differences according to the design and follow up.

In Tunisia, the same BSE, evaluated in our RCT, has already been the subject of two non-comparative studies concerning patients who underwent a total hip replacement [40,41]. The authors concluded that BSE was clinically effective, well tolerated, and free of adverse effects.

The cost difference between BSE ($2.85/day) and BE ($4.5/day) is assessed over a 30-day period. Our research demonstrates that BSE offers a significant cost advantage, with daily savings of $1.65 per patient and a percentage difference of approximately -36.67%. These highlight the economic benefits associated with choosing the BSE option, reinforcing its potential as a cost-effective alternative in clinical practice.

### Predictive factor for thromboses

In this study, there were no predictors of postoperative thrombosis after digestive oncology surgery between the thrombosis and non-thrombosis groups. This was probably due to the relatively low number of thromboembolic events. In the literature, several factors for thrombosis have been determined: patient-related factors, factors related to cancer and associated treatment factors. Indeed, the incidence of thrombosis increases in cancer patients over 80 years, whereas for certain cancers (pancreas, mesothelial tumour, and lung) [42], the incidence of thrombosis was more frequent [42]. The pancreatic cancer had the highest risk of thrombosis [42]. The risk of thrombosis was highest in the first three months after diagnosis of the cancer and decreased with time [42]. Females were at higher risk of venous thrombosis [43]. The combination of three or more comorbidities was a significant predictor of thrombosis in the first year after diagnosis of colorectal cancer [44]. Locally advanced or metastatic colorectal cancer was associated with a higher risk of thrombosis [44]. A retrospective study showed that cancer patients undergoing chemotherapy had a ninefold increased risk of developing thrombosis compared to non-cancer patients [45].

A cohort study, conducted between 2012 and 2016, identified risk factors for thrombosis during hospitalization (advanced age, male gender, steroid use, significant weight loss, preoperative sepsis, prolonged operative time, emergency surgery, and impaired general condition) and risk factors after discharge (steroid use, preoperative sepsis, postoperative complications, and impaired general condition) [23].

As concern BSE safety patients, although the overall mortality rate of 10,1% was high in our study, there was no statistically significant difference between the two groups BSE and BE (12.6% vs 7.4%, p = 0.261). Similarly, there was no statistically significant difference between the two groups with respect to adverse events. The rate of postoperative haemorrhage was 1.1% in the BSE group and 2.5% in the BE group (p = 0.610). The rate of thrombocytopenia was 1.1% in the BSE group and zero percent in the BE group (p = 1.000).

Our RCT placed great importance on its methodology by trying to predefine all the points before starting the trial. However, this RCT had some limitations: 1) It might have been more pragmatic to consider both asymptomatic and symptomatic thrombosis at the same time as primary end point, which would probably have reduced the number of subjects needed to be included. 2) Moreover, the assumption that the primary endpoint would be reached by 18% of patients [8] is most probably not the real rate in Tunisia. However, in practice, there was no prevalence available in our country. So, the trial could be considered underpowered, but in practice the number of subjects needed for inclusion was validated by a methodologist before starting the randomized trial. 3) The immunogenicity study was not initiated at the beginning of the trial. In fact, safety and efficacy studies should show that there were no statistically significant differences between the two treatments regarding their benefits, risks, and the risk of immune reactions [26].

## Supporting information

S1 Checklist CONSORT 2010 checklist of information to include when reporting a randomised trial*. (DOC)

S1 File (DOCX)

S2 File (DOCX)
