# Supplementary material for: Biosimilar versus branded enoxaparin to prevent postoperative venous thromboembolism after surgery for digestive tract cancer: Randomized trial
Source: PLoS One. 2023 Nov 1;18(11):e0293269. doi: 10.1371/journal.pone.0293269 (PMC10619849; doi:10.1371/journal.pone.0293269)
Supplement: S1 File — (DOCX) [file pone.0293269.s002.docx]

**Trial study protocol :**

Comparison of Thromboembolic Events in Patients Undergoing Thromboprophylactic Treatment With ENOXA® vs Lovenox® (ENOXACARE).

[ENOXACARE]

# INTRODUCTION

# Therapeutic area

Thromboprophylaxis in digestive cancer surgery.

# Rationale for the study

Venous thromboembolism (VTE) is defined by the occurrence of deep vein thrombosis (DVT) and / or pulmonary embolism (PE) [1].

The annual incidence of VTE in cancer patients is estimated at 0.5-20% [2], while it is estimated at 0.1% in the general population [3]. It is linked to high morbidity and mortality [4].

In fact, venous thromboembolic disease is the second leading cause of death in cancer patients [5]. ETV is a financial burden on health [6]. This high cost is linked to the following: more consultations, three times more hospitalizations and a longer hospital stay [6].

The high risk of postoperative thrombosis is due to the combination of major risk factors, which are cancer and major surgery [7]. In fact, cancer constitutes a state of acquired hypercoagulability given the multiple relationships between this pathology, the systems of inflammation and hemostasis [8].

Surgery increases the risk of VTE by 29% in the absence of thromboprophylaxis [9]. This is due to vascular damage, immobilization and venous stasis [10]. Multiple risk factors have been identified such as: the nature of the cancer, the stage of the cancer and the associated treatments (radiotherapy, chemotherapy) [11].

Multiple prospective randomized studies done in the early 1980s and late 1990s have shown that thromboprophylaxis decreases the risk of thrombosis compared with no prophylaxis or placebo [12–14]. Similarly, thromboprophylaxis reduced the overall cost of health care [15]. Over the years several molecules have been presented on the market: unfractionated heparin (UFH), low molecular weight heparin (LMWH), antivitamins K (AVK) and direct oral anticoagulants (DOA).

Several guidelines have recommended LMWH or UFH for major abdominopelvic surgery in the absence of risk of bleeding [16–18]. However, LMWH has been the gold standard treatment for cancer-associated TEV [19]. LMWH has advantages over UFH such as: a longer half-life and predictable bioavailability [10].

In addition, LMWH is a less restrictive prescription with only one injection per day compared to two or three injections per day for UFH [16]. A meta-analysis, which included five studies (including 418 patients with cancer), showed a significant reduction in mortality with LMWH compared with UFH [20]. Enoxaparin is a low molecular weight, widely used, safe and effective heparin [21]. Lovenox® and Enoxa® are two enoxaparin treatments. Lovenox® was granted marketing authorization (MA) in 1990 [22]. Enoxa® obtained Marketing Authorization in 2007 [22]. In fact, Enoxa® is less expensive than Lovenox® [23]. The aim of our work was to compare Enoxa® to Lovenox® in patients operated on for digestive cancer with regard to the prevention of the occurrence of venous thrombosis in the post-operative period, to compare the safety of the two treatments and to identify the predictive factors. of a thromboembolic incident.

# STUDY OBJECTIVES

The clinical trial has a primary objective and several secondary objectives.

Primary objective: To compare the incidence of thromboembolic events (symptomatic and asymptomatic) between the two arms of the study, in patients operated on for digestive cancer, and receiving thromboprophylaxis based on enoxaparin sodium.

Secondary objectives:

- Compare the incidence of asymptomatic deep vein thrombosis between the two treatments in the study

- Compare the incidence of symptomatic deep vein thrombosis between the two treatments in the study

- Compare the safety of the two products of the study

- Establish the profile of patients who have developed a thromboembolic incident

# STUDY METHODOLOGY

### Study period

The total duration of the study is 05 years.

### Study location

The study was carried out at the CHU Charles Nicolle (Tunisia), in the ward B of the surgery department.

## Experimental scheme

Comparative study with two arms ENOXA® versus LOVENOX®, randomized, prospective, single-center, with blinded evaluator (rater-blinded).


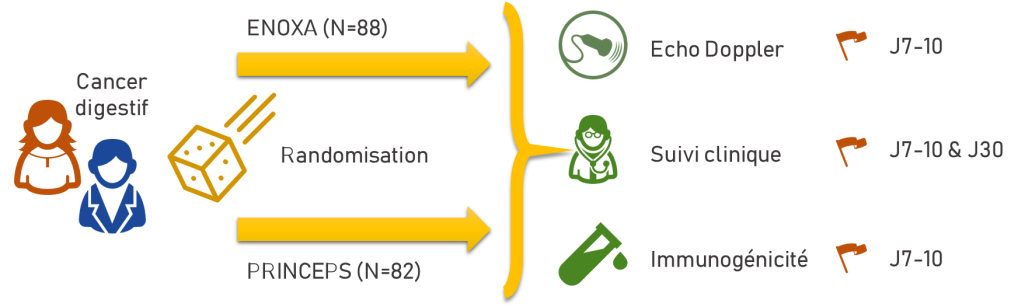


## Study population

Patients with proven, known or newly diagnosed operable digestive cancer requiring enoxaparin thromboprophylaxis at the preventive dosage and admitted for B surgery, in accordance with the following selection criteria:

### 4.3.1 Selection criteria

- Man or woman over 18 years old
- Known or newly diagnosed proven digestive cancer, operable whatever its nature, site or stage
- Emergency or programed surgery patients
- Indication for emergency or programed surgery, whatever its nature
- Preventive administration of enoxaparin sodium

### Non-selection criteria

- Patient participating in another study
- Prior administration with unfractionated heparin in the last 30 days
- Renal failure with creatinine clearance <30 ml / min
- Known history of peripheral and / or deep vein thrombosis occurring in the 3 months prior to inclusion in the study
- Pregnant or breastfeeding woman or of childbearing age not using a medically accepted contraceptive method
- Taking an anticoagulant in the last three months
- Patient with known hemostasis disorder
- Person not wishing to participate in this study, or not having the capacity to understand its objectives

### Exclusion criteria

- Confirmed pregnancy occurring during study follow-up
- Appearance of a secondary contraindication during the study (HIT, severe IR)
- Patients with extra-digestive cancers of the abdomen
- Patients with tumors without histological confirmation.

### Withdrawal of consent

Patients were free to withdraw from the study at any time without giving a reason. Patients were told that if they requested to withdraw from the study, at any time during the trial, there would be no negative consequences.

## Tested products

### Enoxaparin

Depending on the randomization arm, patients will be put on one of the two treatments compared: ENOXA® or LOVENOX®. The administered enoxaparin sodium must comply with the following:

- Administer the dose of 4000 IU per day, regardless of the patient's weight at inclusion

- Start the injections 8 to 12 hours after the operative act

- Administer subcutaneously

- The administration must be daily at a fixed schedule according to clinical practices, for 30 successive days

### Study protocol

The clinical study considered two essential elements

● Step 1: comparative clinical trial Enoxa® versus Lovenox®

This trial has a subclinical event as an endpoint, while taking into account the side effects. This was a study that placed paramount importance on its methodology by trying to predefine all the points before starting the test.

● Step 2: Ancillary arm: immunogenicity study

The immunogenicity study was not initiated from the start of the trial. An amendment to the protocol was introduced along the way, with the objective of evaluating the immunotoxicity profile of the enoxaparin products in the study.

### Randomization

Randomization was established on an open, simple and random 1: 1 allocation. A block allocation of 6 was considered. The allocation list was done automatically DACIMA interface.

### Endpoints

Primary endpoint: Any thromboembolic event, asymptomatic, objectified by Doppler ultrasound of the vessels of the lower limbs on D7 - D10 postoperatively.

Secondary endpoints:

- Occurrence of an asymptomatic venous thrombosis event of the lower limbs on D7 - D10 postoperatively

- Occurrence of a symptomatic thrombotic event

- Occurrence of a TIH incident

- Occurrence of hemorrhagic events

- The volume of intraoperative bleeding

- Occurrence of immunogenicity between the two groups

### Collected Data

- Selection criteria

- Sociodemographic criteria

- Clinical parameters (Temperature, BP, HR, etc.)

- History of the carcinological disease

- Clinical examination on admission and thromboembolic risk factors

- Operative indication and procedure

- Study treatment and concomitant treatments

- Preoperative explorations

- Clinical follow-up and postoperative explorations

- Doppler ultrasound

- Clinical follow-up on D30.

### Organigramme de l’étude

| **Visit** | **Details** |
| --- | --- |
| Eligibility visit | Selection of patients according to inclusion & non-inclusion criteria |
| Initial visit | Signature of informed consent |
| Randomization | Hospital period: period lasting 7 to 10 days postoperatively. Beyond this period, the patient is no longer considered in hospital, even if he is still kept in hospital for whatever reason |
| J7-J10 Control | Random allocation to the product to be tested |
| J30 Control | Ultrasound and clinical control |
| Closing visit | clinical control and study closure |

### Data collection method and personal data protection rules

The data was collected via the DACIMA Clinical Suite® web interface, in accordance with international standards: FDA 21 CFR part 11, HIPPA, ICH, MedDRA and Health Canada and Tunisian regulations.

The DACIMA Clinical Suite® interface provided access to the observation log, which contained all the forms and all the data to be collected from the patient included. The platform is a web interface and does not require any installation. The investigator had to have an internet browser (Chrome, Mozilla Firefox, Internet Explorer, Opera, etc.) and an internet connection. The web address of the electronic logbook was:

**https://secure.dacimasoftware.net/MEDIS**

Access to the DACIMA Clinical Suite® interface was secure and personal. Each investigator used a "username" and a "password" to gain access. Access codes were personal and were not in any way disclosed or delegated to a third party. Each investigator had their own access code.


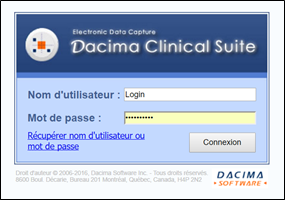


The handing over of the access codes was done on a personal basis. Each investigator was given their own confidential access codes. During the first connection, and for security reasons, the investigator was asked to change his password himself. The new password had to be sufficiently complicated to avoid the risk of hacking: example: contain a number, and a special character (% @?!).


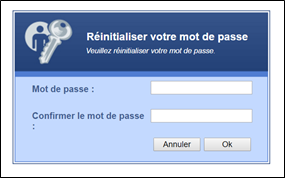


The home page of the DACIMA Clinical Suite® interface contained the list of patients already included a button to add a new patient. The "Randomize a new patient" button allowed you to create a new inclusion. An anonymous and exclusive patient code was generated automatically. Data entry was done by checking boxes or filling in empty fields. Some input fields were hidden and only appeared when specifically activated. For example, the "number of packs / years" only appeared if the investigator checked the "Smoker" box: YES. A demonstration meeting on the use of the electronic case report form was organized with all the investigators, before the start of the inclusions. In addition, the solution presented all the data monitoring tools, in particular by:

● Audit-Trail: Logging and saving of the modifications made to the data

● Queries: exchange and communication between the stakeholders of the study to clarify specific data

● SDV: Source Document Verification Tool

● Lock & Validation: for data review and locking

● Data extraction in Excel & Spss formats.

Personal data protection rules

The sponsor and the investigators have complied with the 2004 organic law relating to the protection of patients' personal data. All CRFs entered on DACIMA Clinical Suite® are anonymous and maintained patient confidentiality.

No personal data of investigators or patients otherwise has been disclosed to any third party.

## 4.5 Audit and data quality

The data collected was verified in order to remedy the missing data. Also controls in the input fields have been introduced to avoid the introduction of outliers. Some required fields have been made mandatory.

## 4.6 Statistical analysis plan and sampling justification

### 4.6.1 Analysis plan

The study data collected is analyzed descriptively at baseline as well as by statistical inference. The study groups are established according to the Enoxa® treatment arm versus Lovenox®.

Continuous parameters are described by the number of valid data, parameter mean, standard deviation, extreme values. The qualitative parameters are described by the number of missing data and valid data, as well as the percentages of the different modalities.

The comparison of the two groups is made for the primary & secondary endpoints. The comparison is made by analysis of variance for paired series for the quantitative parameters and by Mc-Nemar Chi² for the percentages. The distribution of normality for continuous parameters is evaluated by the Kolmogorov-Smirnov test as well as the Shapiro-Wilk test.

The primary endpoint is also assessed by estimating the Confidence Interval of the ratio: test / reference.

The calculation of the primary endpoint is based on the incidence of asymptomatic thromboembolic events objectified by postoperative ultrasound for the two treatment arms.

The secondary endpoints also consider an analysis by treatment group.

A statistical adjustment is made on the independent parameters deemed relevant based on the significance of a logistic regression model.

Statistical analyses were developed independently by the DACIMA Consulting team.

### Sample size

Subclinical thrombosis in postoperative cancer prevention is estimated at 18%. The estimation of the size of the study population is based on the procedure of equivalence of differences in thrombosis frequencies P1 - P2. P1 being the frequency in the ENOXA® group and P2 being the frequency in the comparator group.

The two research hypotheses established for a comparison with the T test

H0 = The difference P1 - P2 ≤ D0L or the difference P1 - P2 ≥ D0U.

H1 = D0L <P1 - P2 <D0U

With D0L = Lower limit of the difference P1 - P2 concluding in an equivalence

With D0U = Highest limit of the difference P1 - P2 concluding in an equivalence

P1 - P2: real difference, with which the power calculation is carried out


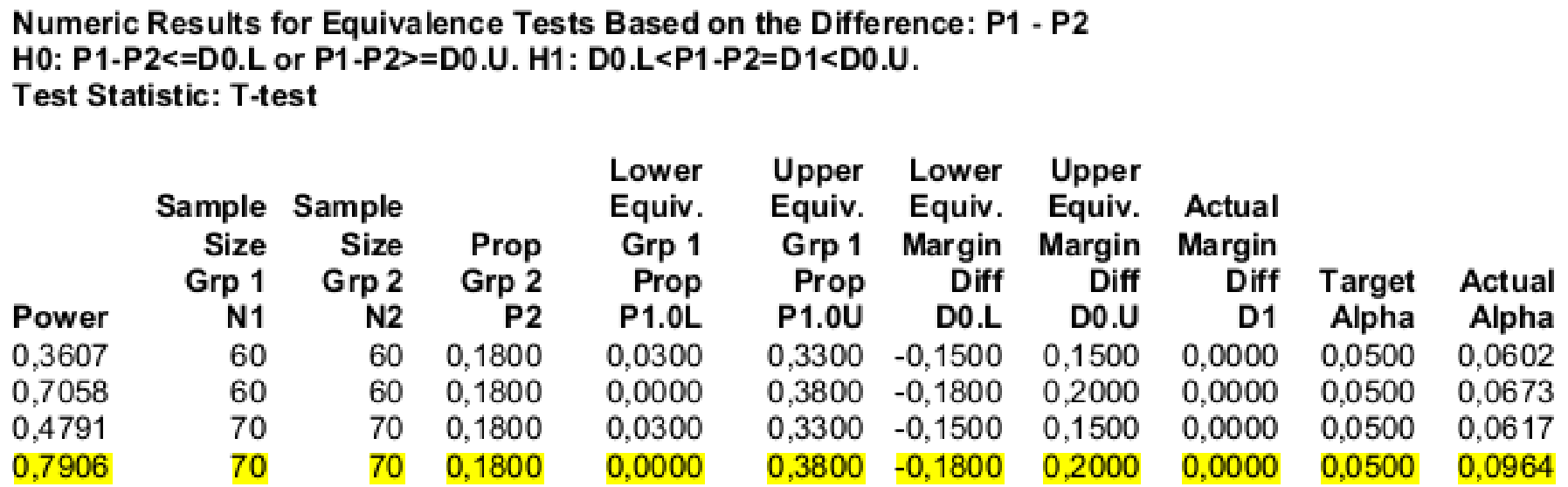


A sample of 70 cases in the test treatment group and a sample of 70 cases in the reference group achieves a power of 0.7906 to detect equivalence. The equivalence margin, estimated in frequency difference, is between -0.18 and +0.2. The actual difference would be 0.00. The calculation assumes the performance of two unilateral Student's T tests. The margin of difference is 0.0964. The sample size estimate is based on the binomial law, used only when the sample size N1 and N2 is less than 100.


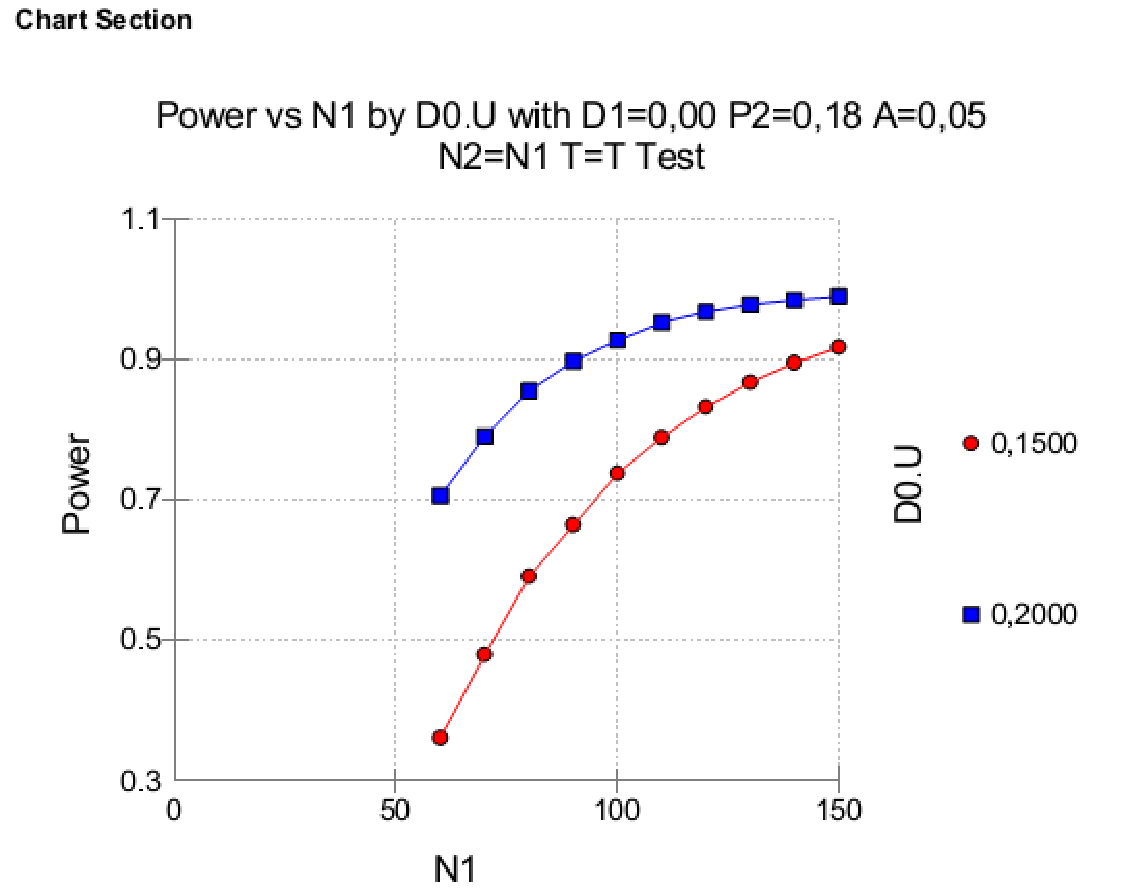


Considering 10% of patients who cannot be analyzed and / or lost to follow-up, a total of 80 patients should be included in each group, or 160 patients in total. The calculation of the sample size involved the PASS v2008 software.

*References*

- *Blackwelder, W.C. 1998. 'Equivalence Trials.' In Encyclopedia of Biostatistics, John Wiley and Sons.New York. Volume 2, 1367-1372.*
- *Chow, S.C. and Liu, J.P. 1999. Design and Analysis of Bioavailability and Bioequivalence Studies. Marcel. Dekker. New York.*
- *Chow, S.C.; Shao, J.; Wang, H. 2003. Sample Size Calculations in Clinical Research. Marcel Dekker. New York.*
- *Farrington, C. P. and Manning, G. 1990. 'Test Statistics and Sample Size Formulae for Comparative Binomial Trials with Null Hypothesis of Non-Zero Risk Difference or Non-Unity Relative Risk.' Statisticsin Medicine, Vol. 9, pages 1447-1454.*
- *Fleiss, J. L., Levin, B., Paik, M.C. 2003. Statistical Methods for Rates and Proportions. Third Edition. John Wiley & Sons. New York.*
- *Gart, John J. and Nam, Jun-mo. 1988. 'Approximate Interval Estimation of the Ratio in Binomial Parameters: A Review and Corrections for Skewness.' Biometrics, Volume 44, Issue 2, 323-338.*
- *Gart, John J. and Nam, Jun-mo. 1990. 'Approximate Interval Estimation of the Difference in Binomial Parameters: Correction for Skewness and Extension to Multiple Tables.' Biometrics, Volume 46, Issue 3, 637-643.*
- *Lachin, John M. 2000. Biostatistical Methods. John Wiley & Sons. New York.*
- *Machin, D., Campbell, M., Fayers, P., and Pinol, A. 1997. Sample Size Tables for Clinical Studies, 2nd Edition. Blackwell Science. Malden, Mass.*
- *Miettinen, O.S. and Nurminen, M. 1985. 'Comparative analysis of two rates.' Statistics in Medicine 4: 213-226.*
- *Tubert-Bitter, P., Manfredi,R., Lellouch, J., Begaud, B. 2000. 'Sample size calculations for risk equivalence testing in pharmacoepidemiology.' Journal of Clinical Epidemiology 53, 1268-1274.*
