# Supplementary material for: Biosimilar versus branded enoxaparin to prevent postoperative venous thromboembolism after surgery for digestive tract cancer: Randomized trial
Source: PLoS One. 2023 Nov 1;18(11):e0293269. doi: 10.1371/journal.pone.0293269 (PMC10619849; doi:10.1371/journal.pone.0293269)
Supplement: S2 File — (DOCX) [file pone.0293269.s003.docx]

**Protocole de l’étude clinique :**

Comparaison des évènements thrombo-emboliques entre EnoxA® et Lovenox® chez les patients opérés pour Cancer digestif et soumis à un traitement thromboprophylactique.

[ENOXACARE]

# INTRODUCTION

# Aire thérapeutique

Thromboprophylaxie dans la chirurgie oncologique digestive

# Rationnel de l’étude

La maladie veineuse thromboembolique (MVTE) est définie par la survenue d’une thrombose veineuse profonde (TVP) et/ou d’une embolie pulmonaire (EP) [1].

L’incidence annuelle de la MVTE chez les patients cancéreux est estimée à 0,5-20% [2], alors qu’elle est estimée à 0,1% chez la population générale [3]. Elle est liée à une morbi-mortalité élevée [4].

En effet la maladie veineuse thrombo-embolique constitue la deuxième cause de décès chez les patients cancéreux [5]. La MVTE constitue un fardeau financier pour la santé [6]. Ce coût élevé est lié aux éléments suivants : plus de consultations, trois fois plus d’hospitalisations et un plus long séjour à l’hôpital [6].

Le risque élevé de thrombose en post-opératoire est dû à l’association de facteurs de risques majeurs qui sont le cancer et la chirurgie lourde [7]. En effet le cancer constitue un état d’hypercoagulabilité acquise vu les multiples rapports entre cette pathologie, les systèmes d’inflammation et de l’hémostase [8].

La chirurgie augmente le risque de la MVTE de 29% en l’absence de thromboprophylaxie [9]. Ceci est dû aux lésions vasculaires, l’immobilisation et la stase veineuse [10]. De multiples facteurs de risque ont été identifiés tels que : la nature du cancer, le stade du cancer et les traitements associés (radiothérapie, chimiothérapie) [11].

De multiples études prospectives randomisées faites au début des années 1980 et fin des années 1990 ont prouvés que la thromboprophylaxie diminuait le risque de thrombose par rapport à l’absence de prophylaxie ou un placebo [12–14]. De même la thromboprophylaxie diminuait le coût global des soins de santé [15]. Au fil des années plusieurs molécules ont été présentées sur le marché : héparine non fractionnée (HNF), héparine à bas poids moléculaire (HBPM), les antivitamines K (AVK) et les anticoagulants oraux directs (AOD).

Plusieurs guidelines ont recommandé l’HBPM ou HNF dans la chirurgie abdomino-pelvienne majeure en l’absence de risque hémorragique [16–18]. Toutefois, l’HBPM a constitué le traitement de référence pour la MVTE associée au cancer [19]. L’HBPM présente des avantages par rapport à l’HNF tel que : une demi-vie plus longue et une biodisponibilité prédictible [10].

En plus l’HBPM constitue une prescription moins contraignante avec une seule injection par jour par rapport à deux ou trois injections par jour pour l’HNF [16]. Une méta-analyse, qui a regroupé cinq études (incluant 418 patients ayant un cancer), a montré une réduction importante de la mortalité avec l’HBPM par rapport à l’HNF [20]. L’énoxaparine est une héparine à bas poids moléculaire, largement utilisée, sûre et efficace [21]. Lovenox® et Enoxa® sont deux traitements à base d’énoxaparine. Le Lovenox® a obtenu l’autorisation de mise sur le marché (AMM) en 1990 [22]. L’Enoxa® a obtenu l’AMM en 2007 [22]. A noter que l’Enoxa® est moins cher que le Lovenox® [23]. Le but de notre travail était de comparer Enoxa® au Lovenox® chez les patients opérés pour un cancer digestif quant à la prévention de la survenue de thrombose veineuse en post-opératoire, de comparer la tolérance des deux traitements et d’identifier les facteurs prédictifs d’un incident thrombo-embolique.

# OBJECTIFS DE L'ÉTUDE

L’essai clinique présente un objectif principal et plusieurs objectifs secondaires

Objectif principal : Comparer l’incidence des évènements thromboemboliques (symptomatiques et asymptomatiques) entre les deux bras de l’étude, chez les patients opérés pour cancer digestif, et bénéficiant d’une thromboprophylaxie à base d’énoxaparine sodique.

Objectifs secondaires :

- Comparer l’incidence des thromboses veineuses profondes asymptomatiques entre les deux traitements de l’étude
- Comparer l’incidence des thromboses veineuses profondes symptomatiques entre les deux traitements de l’étude
- Comparer la tolérance des deux produits de l’étude
- Etablir le profil des patients qui ont développé un incident thromboembolique

# METHODOLOGIE DE L’ETUDE

### Période de l’étude

La durée totale de l’étude est de 05 ans.

### Lieu de déroulement

L’étude a été réalisée au niveau du CHU Charles Nicolle (Tunisie), dans le service de chirurgie digestive Beaux Séjours.

## Schéma expérimental

Etude comparative à deux bras ENOXA® versus LOVENOX®, randomisée, prospective, monocentrique, avec évaluateur en aveugle.


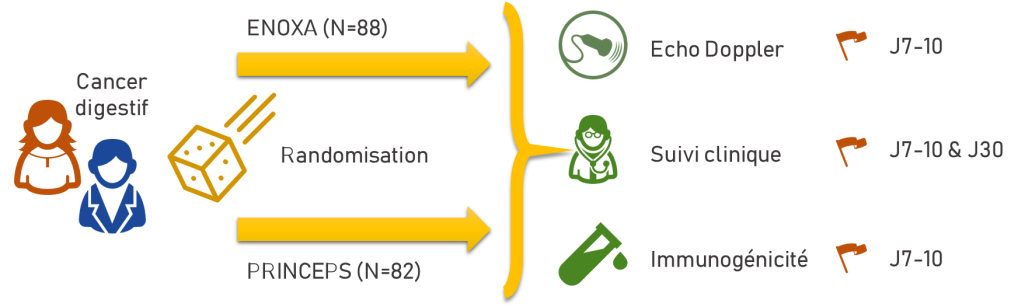


## Population étudiée

Patients présentant un cancer digestif avéré, connu ou nouvellement diagnostiqué, opérable, nécessitant une thromboprophylaxie par énoxaparine au dosage préventif et admis en chirurgie B, conformément aux critères de sélection suivants :

### Critères de sélection

- Homme ou femme ayant plus de 18 ans
- Cancer digestif avéré connu ou nouvellement diagnostiqué, opérable et quel que soit sa nature, quel que soit sa nature, son siège ou son stade
- Patients opérés en urgence ou à froid
- Indication chirurgicale en urgence ou à froid, quel que soit sa nature
- Administration préventive de l’énoxaparine sodique

### Critères de non-sélection

- Patient participant à une autre étude
- Imprégnation préalable à l’héparine non fractionnée dans les 30 derniers jours
- Insuffisance rénale avec clairance de la créatinine < 30 ml/min
- Antécédents connus de thrombose veineuse périphérique et/ou profonde survenue dans les 3 mois précédents l’inclusion dans l’étude
- Femme enceinte ou allaitante ou en âge de procréer n’utilisant pas de méthode contraceptive médicalement acceptée
- Prise d’un anticoagulant dans les trois derniers mois
- Patient avec trouble de l’hémostase connu
- Personne ne désirant pas participer à cette étude, ou n’ayant pas la capacité de comprendre ses objectifs

### Critères d’exclusion

- Grossesse confirmée survenant au cours du suivi de l’étude
- Apparition d’une contre-indication secondaire au cours de l’étude (TIH, IR sévère)
- Patients ayant des cancers extra-digestifs de l’abdomen
- Patients porteurs de tumeurs sans confirmation histologique.

### Retrait de consentement

Les patients étaient libres de se retirer de l'étude à tout moment sans donner de raison. Les patients ont été informés que s'ils demandaient à se retirer de l'étude, à tout moment pendant l'essai, cela n'aurait aucune conséquence négative.

## Produits testés

### Enoxaparine

Selon le bras de randomisation les patients seront mis sous l’un des deux traitements comparés : ENOXA® ou LOVENOX®. L’énoxaparine sodique administrée doit obéir aux éléments suivants :

- Administrer la dose de 4000 UI par jour, quel que soit le poids du patient à l’inclusion des patients
- Démarrer les injections 8 à 12 heures après l’acte opératoire
- Administrer en mode sous-cutané
- L’administration doit être quotidienne à un horaire fixe selon les pratiques cliniques, pendant 30 jours successifs

### Protocole de l’étude

L’étude clinique a considéré deux éléments essentiels

- **Etape 1** : essai clinique comparatif Enoxa® versus Lovenox®

Cet essai a comme critère de jugement un évènement infraclinique, et tout en prenant en considération les effets indésirables. C’était une étude qui a accordé une importance primordiale à sa méthodologie en essayant de prédéfinir tous les points avant d’entamer l’essai.

- **Etape 2**: Bras ancillaire : étude d’immunogénicité

L’étude de l’immunogénicité n’a pas été instaurée dès le début de l’essai. Un amendement au protocole a été instauré en cours de route, dans l’objectif d’évaluer le profil d’immunotoxicité des enoxaparines de l’étude.

### Randomisation

La randomisation a été établie sur une répartition ouverte, simple et aléatoire 1 : 1. Une allocation par Bloc de 6 a été considérée. La liste d’allocation s’est faite de manière automatisée par le logiciel WinPepi version 11.4. La gestion de la randomisation s’est faite par des enveloppes préétablies, scellées et numérotées successivement selon l’ordre chronologique d’inclusion des patients.

### Critères de jugement

Critère principal : Tout évènement thromboembolique, asymptomatique, objectivé par l’échographie Doppler des vaisseaux des membres inférieurs à J7 – J10 post-opératoire

Critères secondaires :

- Survenue d’un événement thrombose veineuse asymptomatique des membres inférieurs à J7 – J10 post-opératoire
- Survenue d’un événement thrombotique symptomatique
- Survenue d’un incident TIH
- Survenue des évènements hémorragiques
- Le volume de saignement per-opératoire
- Survenue d’une immunogénicité entre les deux groupes

### Données collectées

- Critères de sélection
- Critères sociodémographiques
- Paramètres cliniques (Température, TA, FC, etc.)
- Histoire de la maladie carcinologique
- Examen clinique à l’admission et facteurs de risques thromboemboliques
- Indication opératoire et déroulement
- Traitement de l’étude et traitements concomitants
- Explorations préopératoires
- Suivi clinique et explorations postopératoires
- Echographie Doppler
- Suivi clinique à J30.

### Organigramme de l’étude

| **Etape \| Visite** | **Organigramme** |
| --- | --- |
| Eligibilité et visite | Sélection des patients selon les critères d’inclusion & de non-inclusion  Signature du consentement éclairé |
| Visite initiale | Période hospitalière : période durant 7 à 10 jours post-opératoire. Au-delà de ce délai le patient n’est plus considéré en séjour hospitalier, même s’il est gardé encore à l’hôpital pour quel que soit le motif |
| Randomisation | Allocation au hasard au premier inhalateur à tester |
| Contrôle J7-J10 | Contrôle échographique et clinique |
| Contrôle J30 | Contrôle clinique et clôture de l’étude |
| Clôture | Fin de l’étude et clôture |

### Méthode de collecte des données et règles de protection des données personnelles

Les données ont été collectées via l’interface web DACIMA Clinical Suite^®^, conformément aux standards internationaux : FDA 21 CFR part 11, HIPPA, ICH, MedDRA et les réglementations Santé Canada et Tunisienne.

L’interface DACIMA Clinical Suite^®^ a permis l’accès au cahier d’observation, qui contenait tous les formulaires et toutes les données à collecter chez le patient inclus. La plateforme est une interface web, et ne nécessite aucune installation. L’investigateur devait se doter d’un navigateur internet (Chrome, Mozilla Firefox, Internet Explorer, Opera, etc) et d’une connexion internet. L’adresse web du cahier d’observation électronique était :

**https://secure.dacimasoftware.net/MEDIS**

L’accès à l’interface DACIMA Clinical Suite® était sécurisé et personnel. Chaque investigateur a utilisé un « nom d’utilisateur » et un « mot de passe » pour pouvoir y accéder. Les codes d’accès étaient personnels et n’étaient en aucun cas divulgués ou délégués à une tierce personne. Chaque investigateur a eu son propre code d’accès.


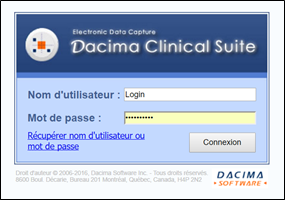


La remise des codes d’accès s’est faite de manière personnelle. Chaque investigateur a reçu ses propres codes d’accès de manière confidentielle. Lors de la première connexion, et par mesure de sécurité, l’investigateur a été invité à modifier lui-même son mot de passe. Le nouveau mot de passe devait être suffisamment compliqué pour éviter le risque de piratage : exemple : contenir un chiffre, et un caractère spécial (% @ ? !).


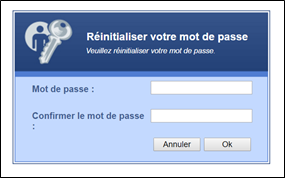


La page d’accueil de l’interface DACIMA Clinical Suite^®^ contenait la liste des patients déjà inclus, un bouton pour ajouter un nouveau patient. Le bouton « **Randomiser un nouveau patient** » permettait de créer une nouvelle inclusion. Un code patient anonyme et exclusif était généré automatiquement. La saisie des données était faite en cochant des cases, ou en remplissant des champs vides. Certains champs de saisie étaient cachés et n’apparaissaient qu’en cas d’activation spécifique. Par exemple, le « nombre des paquets/année » n'apparaissait que si l’investigateur cochait la case « Fumeur » : **OUI**. Une réunion de démonstration de l’utilisation du cahier d’observation électronique a été organisée avec tous les investigateurs, avant le démarrage des inclusions. Par ailleurs la solution présentait tous les outils de monitoring des données, notamment par

- Audit-Trail : Journalisation et sauvegarde des modifications effectuées sur les données
- Requêtes : échange et communication entre les acteurs de l’étude pour clarifier des données spécifiques
- VDS : outil de Vérification des Documents Source
- Verrouillage & Validation : pour la revue des données et verrouillage
- Extraction des données sous formats Excel & SPSS.

Règles de protection des données personnelles

Le promoteur et les investigateurs se sont conforment à la loi organique de 2004 relative à la protection des données personnelles des patients. Tous les CRF saisis sur DACIMA Clinical Suite® sont anonymes et maintenaient la confidentialité des patients.

Aucune donnée personnelle des investigateurs ou des patients par ailleurs n’a été divulguée à aucune tierce partie.

## 4.5 Audit et qualité des données

Les données collectées ont été vérifiées afin de pallier les données manquantes. Également des contrôles dans les champs de saisie ont été instaurés pour éviter l’introduction de données aberrantes. Certains champs requis ont été mis obligatoires.

## 4.6 Plan d’analyse statistique et justificatif d’échantillonnage

### 4.6.1 Plan d’analyse

Les données de l’étude collectées sont analysées de manière descriptive au départ ainsi que par une inférence statistique. Les groupes de l’étude sont établis selon le bras de traitement Enoxa® versus Lovenox®.

Les paramètres continus sont décrits par l’effectif des données valides, la moyenne du paramètre, l’écart-type, les valeurs extrêmes. Les paramètres qualitatifs sont décrits par l’effectif des données manquantes et les données valides, ainsi que les pourcentages des différentes modalités.

La comparaison des deux groupes se fait pour les critères de jugement principal & secondaires. La comparaison se fait par analyse de variance pour séries appariées pour les paramètres quantitatifs et par Chi² de Mc-Nemar pour les pourcentages. La distribution de la normalité pour les paramètres continus est évaluée par le test Kolmogorov-Smirnov ainsi que le test de Shapiro-Wilk.

Le critère de jugement principal est évalué également par estimation de l’Intervalle de Confiance du ratio : test / référence.

Le calcul du critère de jugement principal se fait sur l’incidence des évènements thromboemboliques asymptomatiques objectivés par l’échographie post-opératoire pour les deux bras de traitement.

Les critères de jugements secondaires considèrent également une analyse par groupe de traitement.

Un ajustement statistique est effectué sur les paramètres indépendants jugés pertinents en fonction de la significativité d’un modèle de régression logistique.

Les analyses statistiques ont été élaborées de manière indépendante par l’équipe de DACIMA Consulting

### Taille de l’échantillon

La thrombose infra-clinique en prévention postopératoire carcinologique est estimée à 18%. L’estimation de la taille de la population à étudier repose sur la procédure de l’équivalence des différences des fréquences de thrombose P1 – P2. P1 étant la fréquence chez le groupe ENOXA® et P2 étant la fréquence chez le groupe comparateur.

Les deux hypothèses de recherche établies pour une comparaison avec le test T

H0 = La différence P1 – P2 ≤ D0L ou bien la différence P1 – P2 ≥ D0U.

H1 = D0L < P1 – P2 < D0U

Avec D0L = Limite la plus faible de la différence P1 – P2 concluant à une équivalence

Avec D0U = Limite la plus élevée de la différence P1 – P2 concluant à une équivalence

P1 – P2 : différence réelle, avec laquelle le calcul de la puissance est réalisé.


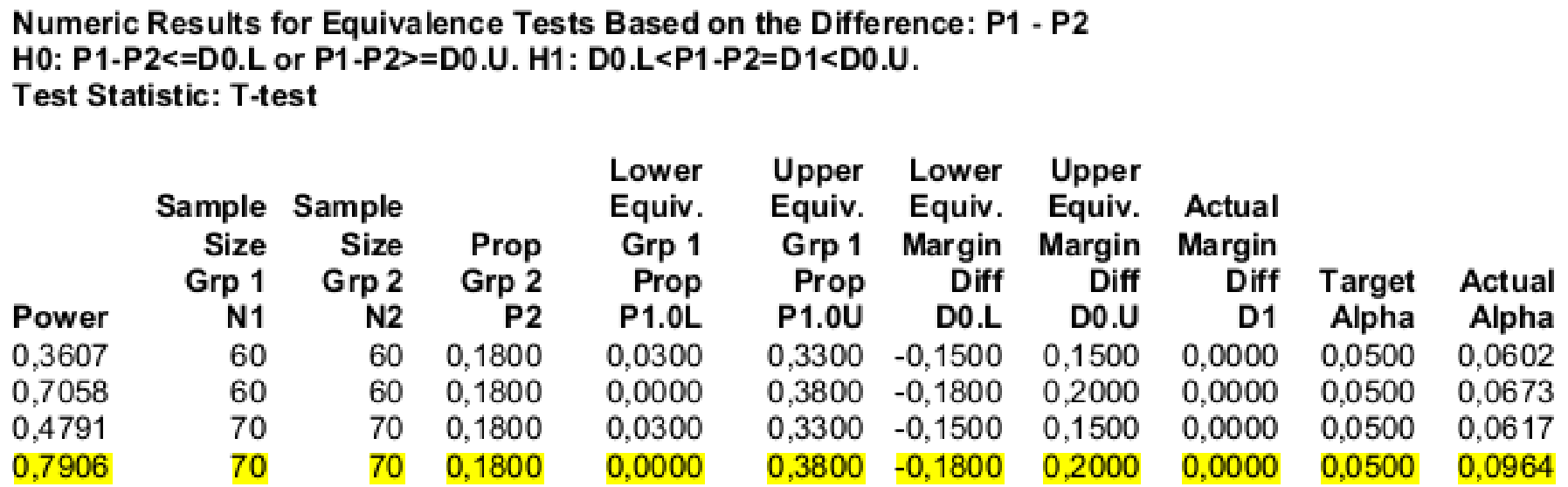


Un échantillon de 70 cas dans le groupe traitement test et un échantillon de 70 cas dans le groupe de référence, réalise une puissance de 0.7906 pour détecter l’équivalence. La marge d’équivalence, estimée en différence de fréquences, est située entre -0.18 et +0.2. La différence réelle serait de 0.00. Le calcul suppose la réalisation de deux tests T de Student unilatéral. La marge de la différence est de 0.0964. L’estimation de la taille de l’échantillon est basée sur la loi binomiale, utilisée uniquement quand la taille des échantillons N1 et N2 est inférieure à 100.


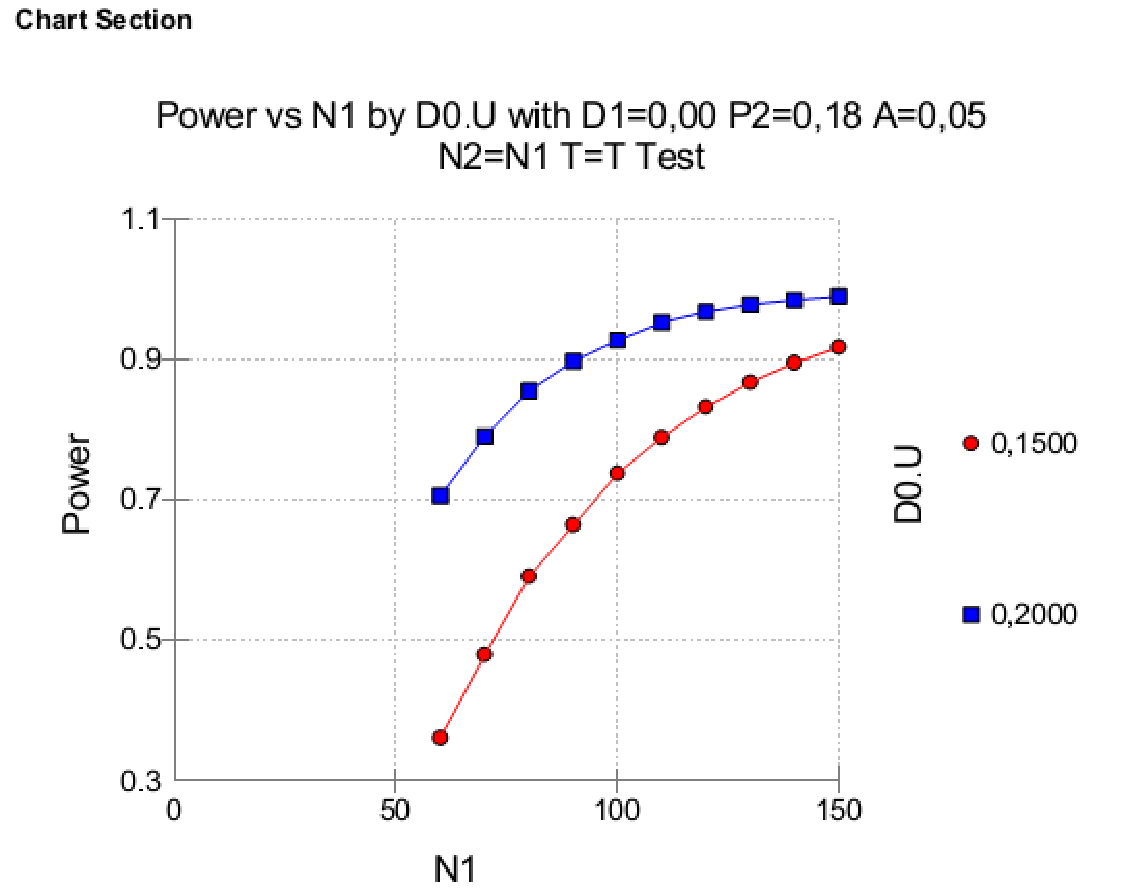


Si l’on considère 10% des patients non analysables et/ou perdus de vue, il faut inclure au total 80 patients dans chaque groupe, soit 160 patients au total. Le calcul de la taille de l’échantillon a fait intervenir le logiciel PASS v2008

*Références*

- *Blackwelder, W.C. 1998. 'Equivalence Trials.' In Encyclopedia of Biostatistics, John Wiley and Sons.New York. Volume 2, 1367-1372.*
- *Chow, S.C. and Liu, J.P. 1999. Design and Analysis of Bioavailability and Bioequivalence Studies. Marcel. Dekker. New York.*
- *Chow, S.C.; Shao, J.; Wang, H. 2003. Sample Size Calculations in Clinical Research. Marcel Dekker. New York.*
- *Farrington, C. P. and Manning, G. 1990. 'Test Statistics and Sample Size Formulae for Comparative Binomial Trials with Null Hypothesis of Non-Zero Risk Difference or Non-Unity Relative Risk.' Statisticsin Medicine, Vol. 9, pages 1447-1454.*
- *Fleiss, J. L., Levin, B., Paik, M.C. 2003. Statistical Methods for Rates and Proportions. Third Edition. John Wiley & Sons. New York.*
- *Gart, John J. and Nam, Jun-mo. 1988. 'Approximate Interval Estimation of the Ratio in Binomial Parameters: A Review and Corrections for Skewness.' Biometrics, Volume 44, Issue 2, 323-338.*
- *Gart, John J. and Nam, Jun-mo. 1990. 'Approximate Interval Estimation of the Difference in Binomial Parameters: Correction for Skewness and Extension to Multiple Tables.' Biometrics, Volume 46, Issue 3, 637-643.*
- *Lachin, John M. 2000. Biostatistical Methods. John Wiley & Sons. New York.*
- *Machin, D., Campbell, M., Fayers, P., and Pinol, A. 1997. Sample Size Tables for Clinical Studies, 2nd Edition. Blackwell Science. Malden, Mass.*
- *Miettinen, O.S. and Nurminen, M. 1985. 'Comparative analysis of two rates.' Statistics in Medicine 4: 213-226.*
- *Tubert-Bitter, P., Manfredi,R., Lellouch, J., Begaud, B. 2000. 'Sample size calculations for risk equivalence testing in pharmacoepidemiology.' Journal of Clinical Epidemiology 53, 1268-1274.*
